# Québec health care professionals’ perspectives on organ donation after medical assistance in dying

**DOI:** 10.1186/s12910-021-00594-7

**Published:** 2021-03-04

**Authors:** Julie Allard, Fabian Ballesteros, Marie-Chantal Fortin

**Affiliations:** 1 Bioethics Program, Department of Social and Preventive Medicine, École de Santé Publique de l’Université de Montréal, Montréal, Canada; 2 Canadian Donation and Transplantation Research Program, Edmonton, Canada; 3 Department of Medicine, Centre de Recherche du Centre Hospitalier de l’Université de Montréal (CHUM), 900, rue Saint-Denis, R12-418, Montréal, QC H2X 0A9 Canada

**Keywords:** Organ donation, Donation after cardiac death, Medical assistance in dying, Qualitative methods, Professionals’ perspectives

## Abstract

**Background:**

Medical assistance in dying (MAID) has been legal in Québec since December 2015 and in the rest of Canada since July 2016. Since then, more than 60 people have donated their organs after MAID. Such donations raise ethical issues about respect of patients’ autonomy, potential pressure to choose MAID, the information given to potential donors, the acceptability of directed donations in such a context and the possibility of death by donation. The objective of this study was to explore Québec professionals’ perspectives on the ethical issues related to organ donation after MAID.

**Methods:**

We conducted semi-directed interviews with 21 health care professionals involved in organ donation such as intensivists and intensive care nurses, operating room nurses, organ donation nurses and coordinators.

**Results:**

The participants were all favourable to organ donation after MAID in order to respect patients’ autonomy. They also favoured informing all potential donors of the possibility of donating organs. They highlighted the importance of assessing donors’ reasons for requesting MAID during the assessment. They were divided on directed donation, living donation before MAID and death by donation.

**Conclusion:**

Organ donation after MAID was widely accepted among the participants, based on the principle of respect for the donor’s autonomy. The findings of this study only provide the perspectives of Québec health care professionals involved in organ donation. Future studies are needed to gather other stakeholders’ perspectives on this issue as well as patients’ and families’ experiences of organ donation after MAID.

## Background

In the Canadian province of Québec, medical aid in dying (MAID) has been legal since December 6, 2015 and consists of the administration of a medication by a physician to patients who meet certain conditions, including capacity to consent, unbearable suffering, serious and incurable illness, and being at the end of life [1]. Organ donation (OD) after MAID is possible for patients with conditions such as neurodegenerative diseases or pulmonary fibrosis and is performed following the protocol of controlled donation after cardiocirculatory death (cDCD). There have been over 70 such donations in Belgium since 2005 and in the Netherlands since 2012 [2–4]. In Canada, from June 2016 to December 2019, more than 60 patients donated organs after MAID [5] (and personal communications). In 2019, 13 donors gave 31 organs to 29 recipients in Québec, accounting for 7% of total donors, while in Ontario there were 19 organ donors (personal communications). Most of the donors suffered from amyotrophic lateral sclerosis [5].

OD after MAID raises many ethical issues as it combines two already ethically challenging procedures [6–12]. Combining MAID and OD raises concerns such as: (i) the importance of patients’ autonomy; (ii) the acceptability of directed donation in such a context, where pressure to save a loved one could push someone to choose MAID in order to donate organs; (iii) the possibility of death by donation in order to raise organ quality and enable heart procurement, which is not currently done through cDCD (i.e. procuring vital organs under anaesthesia and thereby causing death, instead of going through the normal MAID procedure); and (iv) societal pressure, i.e. the possibility that by accepting OD after MAID society creates an underlying duty to choose MAID and donate organs when eligible [6, 11, 12].

In June 2019 Canadian Blood Services (CBS) issued a guidance document for policy on organ donation after MAID [13]. The key points are that patients who consent to MAID should be given the option of donating their organs or tissue, that discussion about OD should take place only after the decision about MAID has been made, and that the guidelines should be reviewed if indications for MAID change. A manual was also published in the Netherlands to guide this emerging practice, but there are still a lot of unanswered questions on the ethical issues outlined above [3]. Even though organ donation after MAID is already performed in Belgium, the Netherlands and Canada, there is no report on the perspectives of health care professionals (HCPs) involved in organ donation. The objective of our study was to explore Québec professionals’ perspectives on the ethical issues related to organ donation after MAID.

## Methods

We conducted an exploratory study on the HCPs’ perspectives using qualitative methods. The interviews took place between November 2016 and February 2018 with HCPs practising in two hospitals selected for their key roles in organ donation and/or transplantation, namely Centre hospitalier de l’Université de Montréal (Saint-Luc and Notre-Dame sites) and Hôpital du Sacré-Coeur de Montréal, and with organ donation coordinators working for Transplant Québec, Québec’s organ donation organization (ODO). Interviews were conducted in French; half were done in person and half over the phone by two different interviewers with experience in qualitative methodology (JA and FB). Except for two participants with whom JA had previous professional interactions, interviewers were not connected to participants before the study.

### Participants

We wanted to reach participants who practiced in services regularly involved in organ donation discussions or organ procurements, such as intensive care unit (ICU) physicians and nurses, surgeons, operation room (OR) nurses and OD nurses/coordinators. At the beginning of the study, since no cases of OD after MAID had taken place and few professionals had experience with MAID in itself, we targeted those participants for their prior knowledge of OD procedures, practical and ethical issues and because they could be involved in organ donation after MAID. In order to recruit HCPs working in the ICU, we presented our project on ICU wards, inviting people to leave their contact information if they were interested in participating. For other HCPs, we used a snowball recruitment technique where recruitment was done through colleagues’ contacts [14]. Thirty-nine people agreed to be contacted. Thirteen individuals (including four surgeons) did not reply to the invitations we sent out, even after two reminders. Five people, two of whom are surgeons, replied that they would participate, but reminders to establish a time for the interview were unsuccessful. Twenty-one participants took part in the study.

### Data collection

We conducted semi-directed interviews. The interview guide was designed from a review of the literature on the ethical issues related to organ donation and MAID [6, 7, 15, 16]. We created vignettes (Table 1) featuring cases raising specific issues, such as directed donation, MAID for mental illness and living donation before MAID. We used vignettes that would describe adults requesting MAID, since it is only available for adult and competent patients in Canada (children and mature minors are not allowed to request MAID at the moment). Vignettes have proven to be useful in qualitative research to clarify peoples’ perceptions and provide a less personal or threatening way of exploring sensitive topics [17]. HCPs were asked to decide whether OD should take place in each clinical situation and give reasons for their decisions. The vignettes were followed by open-ended questions to explore general perspectives on the ethical and practical issues beyond the clinical scenarios of the vignettes. We also asked questions on willingness to participate in procedures, professional experiences with OD after MAID and MAID and socio-demographic issues. The interview guide was tested on three volunteers. Interviews were audio recorded and lasted between 40 and 90 min. Transcripts were not sent to the participants for approval.

**Table 1:** Clinical vignettes

Vignette 1 Mr. A, 39 years old, suffers from an advanced-stage neurodegenerative disease. His pain has become unbearable, and he requests MAID. Given the advanced stage of his disease, he is considered to be at the end of life. His doctor tells him that his request for MAID will be examined and also lets him know that organ donation is a possibility after MAID. Up to that point, Mr. A had not signed his organ donation card nor had he really considered his position on organ donation. However, he is enthusiastic about it and expresses a desire to donate his organs. His MAID request is accepted in accordance with the law His doctor reports the organ donation offer to Transplant Québec. He has no medical contraindications to organ donation In your opinion, should Transplant Québec (i.e., the provincial organ donation organization) agree to retrieve Mr. A’s organs? If yes, why? If no, why not? Are there any other factors that might have altered your decision? What do you think about the doctor having informed the patient about the possibility of organ donation?	
Vignette 2 Ms. B lives in a jurisdiction where MAID for reasons of unbearable suffering is legal outside of the end-of-life context Ms. B, 48 years old, suffers from severe intractable depression. All known treatments have been tried with no improvement in her condition. She no longer wants to live with the suffering and requests MAID. Her doctor, believing she has truly tried every possible option, supports her decision. Her social network is very limited, but her loved ones also support her. Her MAID request is examined and granted. Ms. B expresses a desire to help other sick people by donating her organs after her death. She has no medical contraindications to organ donation Do you think the local organ donation agency should accept Ms. B as an organ donor? If yes, why? If no, why not? Are there any other factors that might have altered your decision? (physical disease vs. psychiatric disease, end of life or not)	
Vignette 3 Mrs. C, 57 years old, suffers from a neurodegenerative disease. She is considered to be at the end of life. She has often expressed the desire to choose the moment of her death and has decided that this moment has come. A niece of Mrs. C suffers from renal failure and is actually on the waiting list to receive a kidney. Mrs. C gathered information on the possibility of donating organs after MAID; she is aware that this process would require her to undergo different medical tests and that she would have to die in the operating room. Mrs. C is willing to go through this process if she is guaranteed that her niece will receive one of her kidneys. If she is compatible with her niece, she agrees to donate the other kidney and other organs (if possible) to patients on the waiting list. If she is not compatible, she renounces organ donation and prefers to die in her room surrounded by her family Her MAID request has been examined and granted in accordance with the law. She has no contraindications to organ donation Do you think Transplant Québec should accept Mrs. C’s organ offer? Why? Are there any factors that could have modified your decision?	
Vignette 4 Mr. D, 53 years old, has a degenerative disease. He plans to request MAID once his suffering becomes unbearable. He has thought about organ donation after MAID but does not like the idea of dying in an operating room. This led him to consider making a living donation before MAID. Since his disease is relatively advanced, he would like to be approved quickly as a living donor so that he can donate a kidney (or maybe even both kidneys, if he can be placed on dialysis for a few weeks) and part of his liver. He is aware that these surgeries could be extremely painful, but he would like to be alive to find out that his organs have saved people, and that they are doing well thanks to him. He thinks this would alleviate his suffering. He also feels that it’s the only useful contribution in years that he’d be able to make to society, so he’d prefer to do it while still alive He has no medical contraindications to organ donation Do you think the living donations team should agree to assess this donor as quickly as possible and proceed with retrieving his organs? What other factors that might have altered your decision?	

### Qualitative analysis

The content of the interviews was transcribed and analyzed by thematic content using NVivo 12 qualitative data analysis software (QSR International) [14]. We mainly used a priori coding, leaving the door open to inductive coding as new themes emerged during the analysis [14]. A first round of coding was done using pre-existing nodes referring to positions on specific ethical issues and on vignettes (in favour of, against, identified reasons for each position). We created new nodes for emerging themes (e.g. respect for autonomy, first-person consent, mental health, discussion on motivations for choosing MAID). Empirical saturation was reached and no new code was created after the 12th interview. A second round of coding was done to capture all data related to the newly created codes. A second coder (FB) coded 20% of the data with the final grid and disagreements were discussed. Coded quotes were then organized by themes and subthemes.

## Results

### Participants

Table 2 presents the participants’ characteristics. Two thirds of participants were nurses, six were ICU physicians and only one was a MAID provider (any physician can be a MAID provider in Québec). All the participants were supportive of OD after MAID. Four had experience related to OD after MAID.

**Table 2:** Characteristics of participants

Characteristics	N = 21 (%)
**Male/female**	7/14 (33.3/66.7)
**Age group**	
21 to 30	3 (14.3)
31 to 40	8 (38.1)
41 to 50	7 (33.3)
Over 50	3 (14.3)
**Profession**	
Nurse [OR/ICU/OD]	14[1/9/4] (66.7)
Intensivist	6 (28.6)
MAID provider	1 (4.8)
**Years of practice**	
0–9	7 (33.3)
10–19	9 (42.9)
20 +	5 (23.8)
**Working site**	
CHUM [Notre-Dame/Saint-Luc]	11 [ 5/6] (52.4)
HSCM	7 (33.3)
Transplant Québec	3 (14.3)
**In favour of OD + MAID**	21 (100)
**In favour of MAID**	19 (90.5)
**Past participation in**	
MAID	2 (9.5)
OD + MAID	4 (19.0)
**Willing to participate in**	
OD + MAID	19 (90.5)
MAID	19 (90.5)

### Themes

#### Respect for autonomy

Nineteen HCPs were in favour of MAID based on the criteria of the prevailing legislation at the time of the interviews (e.g. capacity, unbearable suffering, incurable illness and being at the end of life).

When HCPs were questioned about OD after MAID, they all supported this type of donation, even those who were against MAID. Respect for patient autonomy was the principal reason why participants supported OD after MAID. HCPs underlined that a patient requesting OD after MAID is giving first-person consent and that this is considered the best possible consent, better than most cases of deceased organ donation where families try to determine the donor’s last wishes. Quotes are followed by the numbers identifying the 21 participants and their profession, nurse (N) or physician (P).

If the MAID request is well founded and granted, and on top of it, clearly what the patient wants (…) then it should be even more straightforward, if the patient is truly free to make the decision, I don’t see why he should be refused. P19

But I think, you know, as long as donating his organs is not a motivation for him dying, I don’t know if that’s clear, I think it’s perfectly all right to accept it, because it would be ... um .. respecting people’s autonomy to grant them that, because … I think we have the right to decide what will become of our bodies after our death, you know, we can decide … N14

Yes, absolutely. Because he is conscious, he is able to decide for himself and the idea of donating his organs seems to ease the death. This gentleman has probably used health care in recent years, and I think, philosophically speaking, this gives him something spiritual, it’s very strong, I think for this gentleman to be able to close his eyes and know that other people will benefit from his organs after he dies. N15

Although in favour, two of the HCPs would refuse to participate in OD after MAID: one of them, who is strongly against MAID (P16), refused to be involved in anything related to MAID, and the other participant did not want to deal with the emotional burden associated with OD and MAID (P3). Notably, of the two participants who were against MAID, one would agree to participate in the OD procedure after MAID to honour the patient’s wishes and because he viewed OD as independent from the cause of death.

Yes, I’m not uncomfortable because the MAID decision has been made and it’s nothing to do with me. It’s not that I’m washing my hands of it, but it’s like, make your decisions among adults, you are adults, now you want to hear about organ donations and I can help you with that, but it will not be related to MAID, it will be about what cDCD is, how it works, what happens to the organs. P5

#### Informing patients requesting MAID about OD

When we asked for the participants’ perspectives on the acceptability of informing patients who could be donors and who request MAID of the possibility of donating their organs after MAID, most were in favour, even though the Transplant Québec policy at that time stipulated that the request to donate organs had to come from the patient. A few participants said they were against informing patients: for some of them, Quebec society was not ready for this and we should wait for wider social acceptance of MAID; others feared it could put pressure on patients requesting MAID to opt for OD. These participants nevertheless accepted OD in Vignette 1 where a physician informed his patient upon receiving the MAID request.

For example, participant 3, when asked if he was in favour of informing potential donors, said he was against it, even by way of an information leaflet: “When you give the paper like that you are already putting pressure on. No.” N3.

However, when he studied Vignette 1, he claimed that the information given about OD in the setting of MAID did not pressure the patient, given that in this situation the donor had time to make his own decision, in contrast to deceased organ donation where the decision is made by loved ones who are not necessarily aware of the patients’ wishes and are not ready to make this decision.

Well, when the families … when an accident or something happens, have they thought about it beforehand? Never, never, because it goes faster than that and you are putting that in the hands of someone else, a third party. Look he has the time, it can take a few weeks, a few months, I don’t mind that. N3

Some participants did stress that potential donors should be informed only after the MAID request has been approved, and that discussions regarding MAID and OD should be kept separate. They felt it was important that the manner and timing of such information not be coercive, but rather that information should be provided to give patients the opportunity to fulfil their wishes, another way of respecting patients’ autonomy. Not informing patients about OD was perceived as preventing patients from making an informed choice about their end-of-life care and denying them and their loved ones the psychological benefits associated with OD.

Yes, I think it’s essential; indeed, they need to be informed if they are candidates for organ donation. I wouldn’t want to give a patient false hope. (…) I think the doctor should only mention it if the patient is eligible and everything is in order. N15

It’s any old thing, it’s garbage, it’s wrong to think like that (note: that it should be discussed only if the patient openly requests OD) because that’s like saying that it’s only the patients who know organs can be donated who can, who have access to that. You can’t … you can’t only be entitled to that when you know that it exists. How can you decide to give to this organization or the other when you don’t know such an organization exists? N13

#### Assessment of motivations for MAID

Participants mentioned that the primary motivation for choosing MAID should not be OD but ending the unbearable suffering. They said that motivation for choosing MAID, including OD, and possible pressure for requesting MAID in order to donate organs, should be examined by the physicians evaluating the MAID request.

In the assessment of the MAID request, for example, I think that the motivation for organ donation should be part of the overall evaluation (…) the conditions, the donation really must not be an end in itself. I repeat, medical assistance, I will repeat it often, you will hear me, the purpose of medical assistance for dying is to ease the suffering. Um … that is how it has to stay, it can’t become a way of giving my organs to my neighbour or a friend. It must … ease the suffering. Organ donation can be considered after that, if everything is in place. P11

Well, you know, it’s in relation to his statement, you know, if his motivation is to donate his organs, I mean he only wants to die so that he can donate his organs, I think that would be a problem, but you know, you have to see whether he or she is really at that that point, tired of suffering and doesn’t want to go on living like that, well I think it’s up to him or her to refuse treatments. I think I would look only at those psychological factors. N14

#### Directed donation

When first questioned, some participants were comfortable with donation directed to a specific recipient in the MAID context but others were against it because it goes against the established allocation rules and was seen as unfair.

Organs are allocated by priority based on the waiting list. Well, yes. Unfortunately, I’m for this. N16

No way! (laughs) Mrs. C is really nice, but that’s not how it works! P19

No... it opens the door to too many... patients agree for the cause of organ donation, not to favour a relative. Because I think it isn’t fair for the other patients who are higher than this niece on the waiting list... why wouldn’t they have access when it’s their turn... P7

However, when challenged by the fact that living OD is always directed, most of the participants changed their minds and supported the idea that patients requesting MAID could direct their organs to someone they knew. The next two quotes are from participants who changed their minds about directed OD in the setting of MAID.

You see, my reflex was to say no, but when you think of the living donor context, you don’t have many arguments left to say no... I would still try to explain how values normally guide donation... P7

It would be very sad if someone you knew or someone you wanted to benefit... could not benefit from your own donation. At that point, it’s also justice for the person who wants to donate. Do you need a yes-no answer? Because I’m kind of ambivalent. P19

Although some participants supported directed donation to loved ones, they felt uncomfortable with directed donation to a transplant candidate who publicly solicited OD through the media, primarily because this could prevent patients in urgent need from receiving an organ.

I see discomfort because, in my opinion, there are lists, (…) I would not necessarily agree to giving someone an organ because that person was lucky enough to make the headlines and touch people compared to someone who has not been in the news but is just as sick and needs an organ just as much. That would be … the same way I told you 5 minutes ago that I would agree about her niece, but it doesn’t make sense for me to agree about the niece and agree less about the young girl in the news stories (…) so, in a case like this I am struggling, I am mixed up but I think that, generally speaking, it’s not bad to have lists of recipients and stick to them, because if we don’t it would be … injustice? N17

For some participants, directed donation as a condition for donation was acceptable because it could reduce organ shortages and it is not the health care professionals’ role to decide who receives the organs.

If it’s a *sine qua non* for the patient, it’s the patient who decides to donate, and if she decides one of her organs has to go to a specific person, do we have to judge whether it’s a close relative or not? I think, if that’s her condition for donating, we will get more organs that way. I can’t see any reason for refusing, but I imagine the patient just has to be well informed of everything that is involved, that the patient on TV may jump the line ahead of others who need it more … never mind, just make sure she understands what is involved, but if that is what she decides and we gain one more kidney or lung compared to nothing … You know, in the vignette she seems to be saying that’s her only reason for donating, and as a society we have to grab all the advantages. P10

#### OD and MAID outside the end-of-life context

We explored the participants’ views on OD after MAID outside the end-of-life context for patients with unbearable physical pain with the open-ended questions and for patients with mental illness in Vignette 2. Participants were more comfortable with OD after MAID for the patient with unbearable physical pain than for the patient with mental illness.

Most HCPs were in favour of OD after MAID if the patient was not at the end of life but had physical pain; here there was less uneasiness than with mental illness. A few expressed concerns about the possible consequences of removing the end-of-life criteria to be eligible for MAID.

But, maybe if we say we agree to that, would that push more people to say oh well, I will get MAID in this case … um … yes, that’s it, it opens the door to … lots of things because in my opinion in Law 2, you know, that’s really the end of life but if we open the door to … well, you know if you think you’re worth more dead by giving your organs to someone else, well you can do it. Yes, I’d be afraid of us doing that more and more, it takes away the sacredness of life or, just a collection of organs and we don’t ask any more questions about what we are doing, you know. This is difficult, because on the one hand the person will die anyway, and on the other we’re missing out on organs. N9

For the case of the patient with unbearable depression requesting MAID and OD presented in Vignette 2, opinions were divided. Some were clearly in favour of OD after MAID in this case: either because MAID was legal in the setting of the vignette—underlining that the decision regarding OD was independent of MAID eligibility criteria and they could not see why they would refuse organs from a person who was going to die anyway—or because they saw psychological suffering as a legitimate trigger for MAID.

Yes, absolutely, again, it’s done legally with no undue pressure, the lady is capable of making those decisions. In the worst case, she could do the deed herself in a way that is painful for everyone, she could do the deed in a way that would make her family even more unhappy. For her, to die this way and donate her organs, that’s probably the only way for her to die with dignity, that’s probably the only positive thing in her life and in her passing, both for herself and for her family. N13

Other HCPs expressed that they felt very uncomfortable with MAID in the context of mental illness but that they would ultimately accept OD to respect the autonomy of the patient and for the benefit of transplant candidates on the waiting list.

… I have a hard time accepting that we can’t do anything about someone who wants to die because of their mental suffering. Because, ultimately, that person basically wants to commit suicide, but they can’t… they’re unable to go through with it, and then the government grants them MAID. That’s pretty intense. But as I said, once the MAID request is granted, I don’t see why we couldn’t start the organ donation process, unless the person’s only motivation for MAID is to donate their organs. N14

Lastly, a few HCPs were against OD in the circumstances described in the vignette. Among them, two were strongly opposed to MAID in the case of mental illness because they were not convinced that a patient with a mental illness such as depression was competent to consent to MAID. Another was concerned about undue pressure and black-market organ selling if OD is accepted when there are no end-of-life criteria for MAID, be it for physical or psychological suffering.

No. (…) I’d be afraid of it becoming organ trafficking, when you can get MAID in all kinds of circumstances, as soon as the patient gets a bit depressed, and then you are also legalizing organ donation in that context, personally, where I’m at right now, I would find that a bit more delicate, honestly, I think it would have to be regulated better. P19

#### Living OD before MAID

Views were also divided on the acceptability of the request to donate organs through the living donation procedure before receiving MAID in Vignette 4. Some would have accepted it in order to respect the autonomy of the patient. Most of them specified that the described patient would have to be fully informed of the additional post-surgery suffering, of the ODO’s policy regarding the anonymity of non-directed anonymous donation and of the risk that the outcomes may be disappointing.

I mean, I think it’s OK to do it. I would make sure that he understands what he’s asking for, basically to ensure he’s making an informed decision, hmmm... N14

Other participants were not supportive of living donation before MAID. Some strongly opposed it due to the following considerations for the patient: added suffering, minor benefits and the difficulty of changing their minds about MAID after the surgery. They thought the request was unreasonable and irrational. Others said they would be open to considering the request for only one kidney and if the patient went through the same assessment process as other living donors. They would consider speeding up the process if possible.

I think yes, because it’s like living donations. The patient chooses to donate his organs himself, you know for sure that he will hear about potential recipients, but at the same time we can prepare him by saying he will hear about recipients the same way Transplant Québec tells the families about the patient – he is getting better, he is getting worse, he has left the hospital … Really, I think they are making our lives a bit easier, less complicated because he can give consent before he dies, it’s less uncomfortable. N21

Personally, I wouldn’t feel comfortable assessing him as a living donor, because I don’t think his condition makes him a candidate for living donation. It would only cause his condition to deteriorate and speed up his death, since he’s already close to dying. Then he’d be in even more pain, with almost no quality of life, and we’d be forced to grant MAID earlier than planned because we would have caused additional suffering. P6

Both kidneys—I’d be very uncomfortable with that, because it would have a direct impact on his quality of life, his life expectancy. I would be very uncomfortable with a patient donating both kidneys. That said, we could talk to him about DCD, if he wants to donate a kidney and meets all the donor criteria. I would have no issues with that (…) he would have to go through the process like any other donor. P10

#### Death by donation

We asked participants if they saw a moral difference between procuring organs after the MAID procedure and procuring vital organs under anaesthesia, i.e. death by donation. For many participants, there was confusion between death by donation and living donation before death. When we mentioned the procurement of vital organs under anaesthesia, they referred to their position on Vignette 4 in which the patient wanted to make a living donation before proceeding with MAID. We reasserted that this case was different as the patient would die from the donation, but some participants still mentioned that the patient would return to his room and die right after the procurement. Out of respect for these participants, we did not try to explain the concept a third time.

We restricted our analysis to the 13 participants for whom the concept of death by donation was very clear. Among these participants, a few were in favour of death by donation, stating that it was hypocritical to wait for the death of the patient when it was medically induced anyway.

It’s the same thing, there’s no moral difference between killing a patient, then taking their organs, and killing a patient by taking their organs (laughs). No, there’s no difference, they’re dead either way. N3

There has been full compliance with the MAID procedure, so, technically, whether it’s done by injection or by anaesthesia and then removing the heart, I don’t see a problem. But of course, it’s going to be an issue for some religious groups. It’s going to be a matter of perception. N9

Many participants did not see a moral difference between causing death by MAID and procuring organs afterwards and procuring vital organs under anaesthesia, which would cause the death of the donor. However, they thought that this practice could be deleterious to the perception of OD, evoking the vulture image. They thought that the risks of affecting public trust were more significant than the benefits that could be obtained from the quality of the organs.

Is there a big difference, morally speaking? Perhaps not so much, except for, once again, the image that it projects, that it’s the organ retrieval that’s killing him…honestly. (…) ultimately, this doesn’t change anything for the patient, because he won’t suffer, won’t feel anything… In fact, the warm ischemia time will probably be shorter—no, it will definitely be shorter—but I’m not sure it’s worth the trouble. Given the image it projects, personally, I would be completely opposed to it. P10

A few were strongly against the idea without offering further justification beyond being uncomfortable with it. Those participants saw a moral difference between death by donation and OD after MAID.

Oh my God! No, no, no! That’s absolutely not done. No way! That’s just not done in Québec. There’s absolutely no way. You can’t decide to donate your organs before you die, and you especially can’t decide to donate your organs as a way of dying. It’s just not done! N13

#### Conscientious objection

The question of conscientious objection to OD after MAID was brought to us during the data-collection process. An open-ended question on this issue was added for the last 10 participants. Only one participant was against conscientious objection from HCPs in the OD and MAID context, saying that HCPs have a job to do and that they must do it.

I don’t think there’s any place for conscientious objection. Not by anyone, any professional. We have a job to do, and we need to do it. N13

All other participants were in favour of respecting conscientious objection from any person involved in the process. Many underlined that objections may arise from misunderstanding or myths surrounding the procedure and that discussion and training were important for dispelling any misconceptions. If the unease still persists after being well informed, they emphasized that it is important that all HCPs involved be at ease, that this is necessary to ensure good care of the patient and respect the patient, who needs to be in a peaceful setting.

I think anyone might have a conscientious objection about this topic, because even though we’re health care professionals, everyone has their own values, opinions, religious beliefs, past histories, and experiences. So, I think anyone might have a conscientious objection about this, and I think it’s important to respect that. N17

## Discussion

At the time of data collection, the only guidelines available to Québec HCPs were the position statements published by the Transplant Québec Ethics Committee and the Commission de l’éthique en science et technologie (CEST), both in favour of OD after MAID in order to respect patient autonomy [18, 19]. A Dutch practical manual was also published [3]. Since then, the group led by Canadian Blood Services published the organization’s guidance document for policy, [13] and the Dutch Transplant Foundation published its national guidelines on organ donation after euthanasia [20]. Table 3 summarizes the available guidelines and the participants’ perspectives on the main ethical issues. The participants’ views were mostly in line with published recommendations on issues such as respect for autonomy, the duty to inform MAID patients about the possibility of donating organs after the MAID request has been granted (except for the Dutch recommendation), living donation before MAID and conscientious objection. Participants who understood the concept of death by donation also had views aligned with published recommendations. That many participants did not understand the procedure of death by donation may reflect the fact that the procedure contravenes the dead-donor rule, which is widely accepted in the OD community. It is possible they could not conceive that we were referring to a procedure that would go against this rule. Further research is needed to explore this concept and deepen the analysis of HCPs’ perspectives. We will more fully discuss issues where there are divergences or where HCPs’ views raised new questions.

**Table 3:** Comparison of recommendations on different issues and HCPs’ perspectives

	Canadian Blood Services [13]	Transplant Québec Ethics Committee [18]	CEST(19)	Dutch national guidelines [20]	Participants’ perspectives
Informing potential donor	All eligible, medically suitable patients should be given an opportunity to consider organ and tissue donation Discussions concerning donation should happen only after patients have been found eligible for MAID by 2 independent assessments	2016: Respond only to patient-initiated requests 2018: All MAID patients eligible for OD should be informed about the possibility of donating organs Discussions concerning OD should take place after MAID request has been accepted. OD as a motivation for MAID has to be assessed by the physician assessing the MAID request	All patients eligible for MAID should be informed of the possibility of donating organs after MAID and of the effects on their end of life Strict separation of discussions about MAID and OD	Physicians should not inform potential donors. Requests for organ donation should be initiated by the patient who asked for euthanasia Separation of discussions is not mentioned. The treating physician is responsible informing the patient about OD and ensuring that the autonomy of the patient is safeguarded	In favour of informing all MAID patients who are eligible for OD Discussions concerning OD should take place after MAID request has been granted. OD as a motivation for MAID has to be assessed
Directed donation	Should not be offered or encouraged, but should be examined on a case-by-case basis if a patient insists	Not mentioned Transplant Quebec has accepted deceased directed donation before MAID was practised	Not mentioned	Not mentioned as it is not permitted in the Netherlands	In favour after comparing with living donation
Living organ donation before MAID	Should not be offered or encouraged, but should be examined on a case-by-case basis if a patient insists	Not mentioned	Not mentioned	Not mentioned	Divided positions. Some participants against because of added suffering and minor benefits. Others in favour of assessing the case
Death by donation	The dead-donor rule must always be respected. Vital organs can be procured only from a deceased donor; the act of procurement cannot be the immediate cause of death	Recommendation to follow the normal cDCD procedure and respect the dead-donor rule	Not openly mentioned, but strict separation of the procedures and teams to preserve public trust is mentioned	Not mentioned	Most participants were either against the practice per se or in order to protect public trust in OD
Conscientious objection	HCPs can object to MAID but their objection should not impede the ability of the patient to donate. Participation of HCPs should be voluntary when possible	Not mentioned	Possible moral distress of HCPs is mentioned without taking a position on the will to participate in the procedure	Hospitals should deal prudently with care professionals not wishing to become involved in euthanasia as a matter of principle, and replace them with colleagues who want to be involved in the procedure on a voluntary basis	All but one participants were in favour of respecting conscientious objection and deploying only willing HCPs
OD and MAID without the end-of-life criteria	Recommendations would have to be reviewed if indications for MAID changes	Not mentioned	Not mentioned	Already practised, no difference mentioned	Participants are divided about OD in this context

### Separation of discussion about MAID and discussion about OD

Except for the position of the Dutch Transplant Foundation stipulating that the OD request should be initiated by the patient, other recommendations point toward informing all MAID patients who qualify for OD once the MAID request has been granted. However, at the time of the interviews, the local recommendation was not to inform potential donors, but to proceed only with patients who had initiated the OD request. Some participants, interviewed a few months after MAID became legal, also thought it would be better to wait for MAID acceptance by the population and reassess after a few years. It is noteworthy that few participants thought we should not inform potential donors of the possibility of donating organs as it could pressure them into donating organs, but saw no problem in Vignette 1 where the physician informs the patient about OD when he makes his MAID request. Even though some pointed out that it should have been done after the MAID request was granted, all participants thought it was acceptable to accept the organs of Mr A who had been informed by his physician about the possibility of donating his organs. This contradiction shows that theoretical positions on an issue may differ from what is perceived as right in the clinical context, and underlines the importance of exploring views with clinical vignettes.

Recommendations are consensual regarding the necessity of maintaining a strict separation between discussions about MAID and discussions about OD. However, we noted a contradiction between this recommendation and the will expressed by both participants and the CBS guidance document to prevent OD from being a motivation for MAID. When participants discussed specific clinical scenarios, they often mentioned the need to explore motivations and possible pressure to choose MAID for OD during the MAID request assessment, particularly in cases of directed donation or outside of the end-of-life context. The Transplant Québec Ethics Committee states that OD as a motivation has to be assessed by the physicians assessing the MAID request, while also recommending separation of discussions [18]. The CBS’s guidance document for policy states that the main ethical concern about OD after MAID is that the decision for MAID could be driven by a desire to donate organs. CBS suggests postponing any discussion about OD to prevent the desire to donate organs from being the motivation for MAID [13]. Postponing the discussion about organ donation with a patient who raises the subject before the MAID request has been granted will, however, deprive physicians of the opportunity of exploring the importance of OD in the decision-making process.

### Directed donation

Directed donation is one of the most controversial issues of OD after MAID [13]. The Appendix of the CBS guidance document for policy reports that most experts participating in the forum felt great discomfort about directed donation, but they also felt discomfort at the thought that forbidding it may push patients to choose living donation before MAID in order to be able to direct their donations, thus causing more pain and hurdles for patients [21]. The province of Saskatchewan has forbidden directed donation explicitly in its policy on MAID [22]. The practice is also not permitted in Belgium and the Netherlands [3]. The Transplant Québec Ethics Committee does not mention directed donation in relation to MAID but accepts deceased directed donation under specific conditions [23]. Although many participants were at first reluctant, they thought patients should be able to decide to whom their organs would go, just as living donors do. Their primary reaction, the same as that of the experts consulted by the CBS, could be linked to their in-depth knowledge of deceased donor organ allocation schemes. Interviewing experts on living OD may have yielded different results, since they are used to seeing patients who are willing to go through specific medical procedures for a specific recipient. The fact that our participants’ perspectives changed during the interviews invites a broader discussion about the ethical issues associated with directed OD in the MAID context in which we could further develop policies. Special consideration should also be given to directed donation to a transplant candidate who publicly solicited organ donation, given that HCPs were divided on this issue.

### OD after MAID outside the end-of-life context

Most of our participants were favourable to OD in a context where MAID is given without the end-of-life criteria, although a few were against it in the case of mental illness, which was not covered by Transplant Québec, CEST or CBS because MAID was not permissible for mentally ill patients at the time of publication. MAID outside of the end-of-life criteria is accepted in the Netherlands and Belgium; to our knowledge, differences between OD in the two contexts has not been discussed. The views of Québec’s HCPs on this topic are important because the end-of-life criterion was removed in March 2020 in Québec following a ruling by the Superior Court of Québec [24]. Further studies and consultations are necessary to design guidelines and safeguards for this new context, as well as for OD after MAID in the case of mental illness. The participants’ unease with MAID in this context reflects the ongoing debates on the acceptability of MAID in this context and the conditions under which it could be acceptable [25–29]. There will be wide public consultation on these issues in Quebec [30].

## Limitations

Our study has limitations. First, we were not able to collect surgeons’ views even though some agreed to participate in our research. The participants are from the metropolitan Montréal area and results may not reflect views from other regions or provinces. The fact that participants registered themselves could have led to a selection bias of people in favour of the procedure. Because interviews were conducted over an 18-month period, there could have been an impact on the acceptance of MAID by HCPs, however, only one of the first 12 participants was against MAID, and specified that he would not change his position on this in the next 20 years; he was not against assisted death in itself but against assigning it to physicians, since it was in conflict with their duty to save patients. There was no change in legislation or guidelines during this time period. We interviewed only one MAID provider and only a few of our participants had experience with the MAID procedure or with OD after MAID, as only a few cases of OD after MAID had occurred by the end of our data collection period. Despite these limitations, this is the first study to report HCPs’ perspectives on OD and MAID and the findings could be useful for future studies.

## Conclusion

All our participants underlined the importance of respecting patients’ wishes whenever possible, once the patients have been fully informed of all the consequences of their choices and if there is no foreseeable negative impact on OD. Exploring the perspectives of HCPs with clinical vignettes on issues associated with OD after MAID gave us insight on how the ethical issues may be perceived in the clinical setting. It highlights the fact that sound ethical analysis is needed for assessing the motivation for MAID, the acceptability of directed donation for a relative or a stranger and the acceptability of OD after MAID when the end-of-life criterion is removed. Further studies are needed to explore how the procedure is experienced by HCPs, patients and families.

## Data Availability

The datasets used and/or analysed during the current study are available from the corresponding author on reasonable request.

## Abbreviations

CBS: Canadian Blood Services
cDCD: Controlled donation after cardiocirculatory death
CEST: Commission de l’éthique en science et technologie
CHUM: Centre hospitalier de l’Université de Montréal
HCPs: Health care professionals
HSCM: Hôpital Sacré-Coeur de Montréal
ICU: Intensive care unit
MAID: Medical assistance in dying
N: Nurse
OD: Organ donation
ODO: Organ donation organization
OR: Operation room
P: Physician
